# Global trends and research gaps in deep brain stimulation for neurodevelopmental disorders: a scientometrics exploration of functional and behavioral outcomes

**DOI:** 10.3389/fnhum.2025.1649726

**Published:** 2025-08-29

**Authors:** Mónica Acuña-Rodriguez, Kevin Fernando Montoya-Quintero, Fabriccio J. Visconti-Lopez, Judith Cristina Martinez-Royert

**Affiliations:** ^1^Universidad de la Costa, Barranquilla, Colombia; ^2^Facultad de Ciencias para la Salud, Universidad de Manizales, Manizales, Colombia; ^3^Universidad Científica Del Sur, Lima, Peru; ^4^Facultad de Ciencias de la Salud, Centro de Investigaciones en Ciencias de la Vida, Universidad Simón Bolívar, Barranquilla, Colombia

**Keywords:** deep brain stimulation, neurodevelopmental disorders, evidence gaps, neurosciences, knowledge discovery

## Abstract

**Introduction:**

Deep brain stimulation has emerged as a potential intervention for improving functional outcomes and behavioral regulation in individuals with neurodevelopmental disorders. However, the extent to which these clinical endpoints have been systematically studied remains unclear.

**Methods:**

A comparative scientometrics analysis was conducted to map the research landscape on deep brain stimulation in neurodevelopmental disorders (*n* = 833 publications) and a focused subset addressing functional performance and aggressiveness (*n* = 52). We used Bibliometrix in R and Matplotlib in Python to analyze studies published between 1996 and June 2025.

**Results:**

We found a sustained increase in publication volume since 1996, but only 6.2% of studies explicitly addressed functional or behavioral endpoints. Clinical trials and systematic reviews were underrepresented in both datasets (1.20 and 3.12% in the general analysis; 0 and 7.69% in the subset, respectively). High-income countries dominated scientific production, with minimal contributions from lower-income regions. In the focused subset, Colombia emerged among the top 3 most productive countries. Keyword analyses revealed a thematic concentration on diagnostic categories rather than outcome-based evidence.

**Discussion:**

These findings highlight critical research gaps and suggest a misalignment between current scientific focus and the clinical potential of deep brain stimulation in neurodevelopmental disorders. Future studies should focus on functional improvement and behavioral modulation to bridge this divide and support the development of evidence-based, patient-centered applications of deep brain stimulation in individuals with neurodevelopmental disorders.

## 1 Introduction

Deep brain stimulation (DBS) has emerged as a promising therapeutic intervention in a variety of neuropsychiatric conditions (Krauss et al., 2021), offering potential benefits in domains traditionally considered difficult to treat pharmacologically or behaviorally (Krauss et al., 2021). Among these, neurodevelopmental disorders represent a particularly complex frontier (Chen et al., 2024), not only due to their heterogeneous presentations and early onset but also because of the chronic nature and multifactorial etiology that underlie functional impairments and behavioral dysregulation (Chen et al., 2024). In recent years, DBS has been increasingly explored as a strategy to improve adaptive functioning and reduce pathological aggressiveness in this population, signaling a paradigm shift from symptomatic control to functional restoration (Tremblay- McGaw et al., 2025).

Despite this growing clinical interest, little is known about how the scientific landscape has evolved in this domain. Specifically, there is a lack of systematic understanding of the volume, focus, and direction of research efforts that have sought to connect DBS with neurodevelopmental disorders, let alone those targeting key outcomes such as functional performance and behavioral regulation (Liu et al., 2022). Such knowledge is crucial not only to map current evidence but also to identify underexplored areas, theoretical, knowledge or methodological gaps, and opportunities for innovation in both clinical and translational neuroscience (Lozada-Martinez et al., 2025a,b; Lozada-Martinez et al., 2024).

This study does not evaluate clinical effectiveness but rather maps the global research trends through scientometrics methods. Previous efforts have partially explored the topic from a clinical standpoint (Chen et al., 2024; Tremblay- McGaw et al., 2025), but a comprehensive bibliometric perspective remains lacking. The absence of such analysis constitutes a methodological blind spot in the literature, particularly given the increasing need for data-informed strategies to prioritize research funding, clinical trials, and collaborative efforts across disciplines (Lozada-Martinez et al., 2025a,b).

In this review, we address this gap by conducting a dual-layered scientometrics analysis. First, we explore the overall scientific output connecting DBS with neurodevelopmental disorders over the past three decades. Second, we focus specifically on studies linking DBS to functional performance and aggressiveness, with the aim of identifying knowledge gaps and underrepresented research themes. Our findings offer a meta-research lens through which to assess the current state of the field and illuminate strategic directions for future investigation and clinical translation.

## 2 Methods

A comprehensive and systematic search according to PRISMA guideline (Page et al., 2021) was conducted across three major scientific databases: Scopus, Web of Science Core Collection, and PubMed, to capture the broadest possible representation of the global scientific output on DBS in neurodevelopmental disorders. The search included all indexed records up to June 12 2025, with no restrictions on language or country of origin.

The search strategy was designed using controlled vocabulary (MeSH terms in PubMed) and free-text terms, based on validated descriptors and expert consultation. Synonyms and variations were incorporated for all key concepts, including DBS (MeSH Unique ID: D046690), neurodevelopmental disorders (MeSH Unique ID: D065886), physical functional performance (MeSH Unique ID: D000076604), and aggression (MeSH Unique ID: D000374). Boolean operators, truncation rules and field tags were adapted for each database to ensure consistency across platforms.

As an example, the following search strings were used in Scopus to retrieve the datasets included in this study:

- General search (DBS and neurodevelopmental disorders): TITLE-ABS-KEY (“deep brain stimulation*” OR “electrical stimulation of the brain” AND NOT rat OR mouse OR mice OR rodent OR “animal model*”) AND TITLE-ABS-KEY (“neurodevelopmental disorder*” OR “mental disorder* usually diagnosed in infancy” OR “mental disorder* diagnosed in childhood” OR “child mental disorder*” OR “child behavior disorder*” OR “pervasive child development disorder*” OR “developmental disabilit*” OR “intellectual disabilit*” OR “learning disabilit*” OR “specific learning disorder” OR “speech sound disorder” OR “communication disorder*” OR “social communication disorder” OR “childhood-onset fluency disorder” OR “autism spectrum disorder” OR “reactive attachment disorder” OR “mutism” OR “sluggish cognitive tempo” OR “oppositional defiant disorder” OR “conduct disorder” OR “attention deficit disorder with hyperactivity” OR “attention deficit and disruptive behavior disorder*” OR “separation anxiety” OR “motor skills disorder*” OR “tic disorder*” OR “tourette syndrome” OR “stereotypic movement disorder” OR “childhood schizophrenia”).
- Focused sub-analysis (including functional performance and aggressiveness): TITLE-ABS-KEY (“deep brain stimulation*” OR “electrical stimulation of the brain” AND NOT rat OR mouse OR mice OR rodent OR “animal model*”) AND TITLE-ABS-KEY (“neurodevelopmental disorder*” OR “mental disorder* usually diagnosed in infancy” OR “mental disorder* diagnosed in childhood” OR “child mental disorder*” OR “child behavior disorder*” OR “pervasive child development disorder*” OR “developmental disabilit*” OR “intellectual disabilit*” OR “learning disabilit*” OR “specific learning disorder” OR “speech sound disorder” OR “communication disorder*” OR “social communication disorder” OR “childhood-onset fluency disorder” OR “autism spectrum disorder” OR “reactive attachment disorder” OR “mutism” OR “sluggish cognitive tempo” OR “oppositional defiant disorder” OR “conduct disorder” OR “attention deficit disorder with hyperactivity” OR “attention deficit and disruptive behavior disorder*” OR “separation anxiety” OR “motor skills disorder*” OR “tic disorder*” OR “tourette syndrome” OR “stereotypic movement disorder” OR “childhood schizophrenia”) AND [TITLE-ABS-KEY (“physical functional performance” OR “physical functional performances” OR “functional performance” OR “functional performances” OR “physical performance” OR “physical performances”) OR TITLE-ABS-KEY (aggression OR aggressiveness)].

Records retrieved from the three databases were merged, and duplicates were removed manually and through automated filtering in Microsoft Excel (version 2019). Conference papers, errata, early access articles, books, book chapters, book series, and non-peer-reviewed materials were excluded. Data extraction and standardization were independently performed and cross-validated by two researchers to ensure accuracy and consistency. Discrepancies were resolved through consensus.

From the final dataset, the following bibliographic metadata were extracted: title, year of publication, journal name, citation count, author keywords, funding source, country of the corresponding author, geographic region, income group, language, and document type. These fields were selected to facilitate multidimensional scientometrics analysis encompassing productivity, impact, and thematic focus. All data were manually verified and standardized to ensure consistency, particularly in fields such as keywords, country names, and institutional affiliations.

The variables were defined as follows: “Funding” referred to whether the publication explicitly reported financial support from any institution or grant. “Study type” was categorized based on document type into original articles, reviews, and others. “Impact” was operationalized using citation count as a proxy indicator.

The countries of the corresponding authors were additionally classified according to geographic regions and income groups, following the most recent classification published by The World Bank (2024). This enabled stratified analyses of global contributions and research equity across different economic and geographic contexts.

The analysis was conducted in two phases:

1. A general analysis of the full body of literature on DBS in neurodevelopmental disorders.
2. A focused sub-analysis on the subset of studies explicitly addressing functional performance and/or aggressiveness within the same context.

Descriptive statistical analyses were performed using the R programming language (version 4.3.1) (Chan, 2018), employing the Bibliometrix package and base R functionalities. Matplotlib in Python (version 3.9) were used for visualization. Frequencies and percentages were computed for all categorical variables. Publication and citation trends were visualized using combined bar-line and area charts styled for clarity and interpretability. Word frequency and co-occurrence analyses were performed on the author keywords to explore thematic evolution and identify research hotspots.

This study involved only publicly available bibliometric data and did not include human subjects, biological samples, or any individual-level information. Therefore, ethical approval from an institutional review board or ethics committee was not required.

## 3 Scientific output on DBS in neurodevelopmental disorders

### 3.1 Baseline characteristics and publication trends

**Figure 1:**
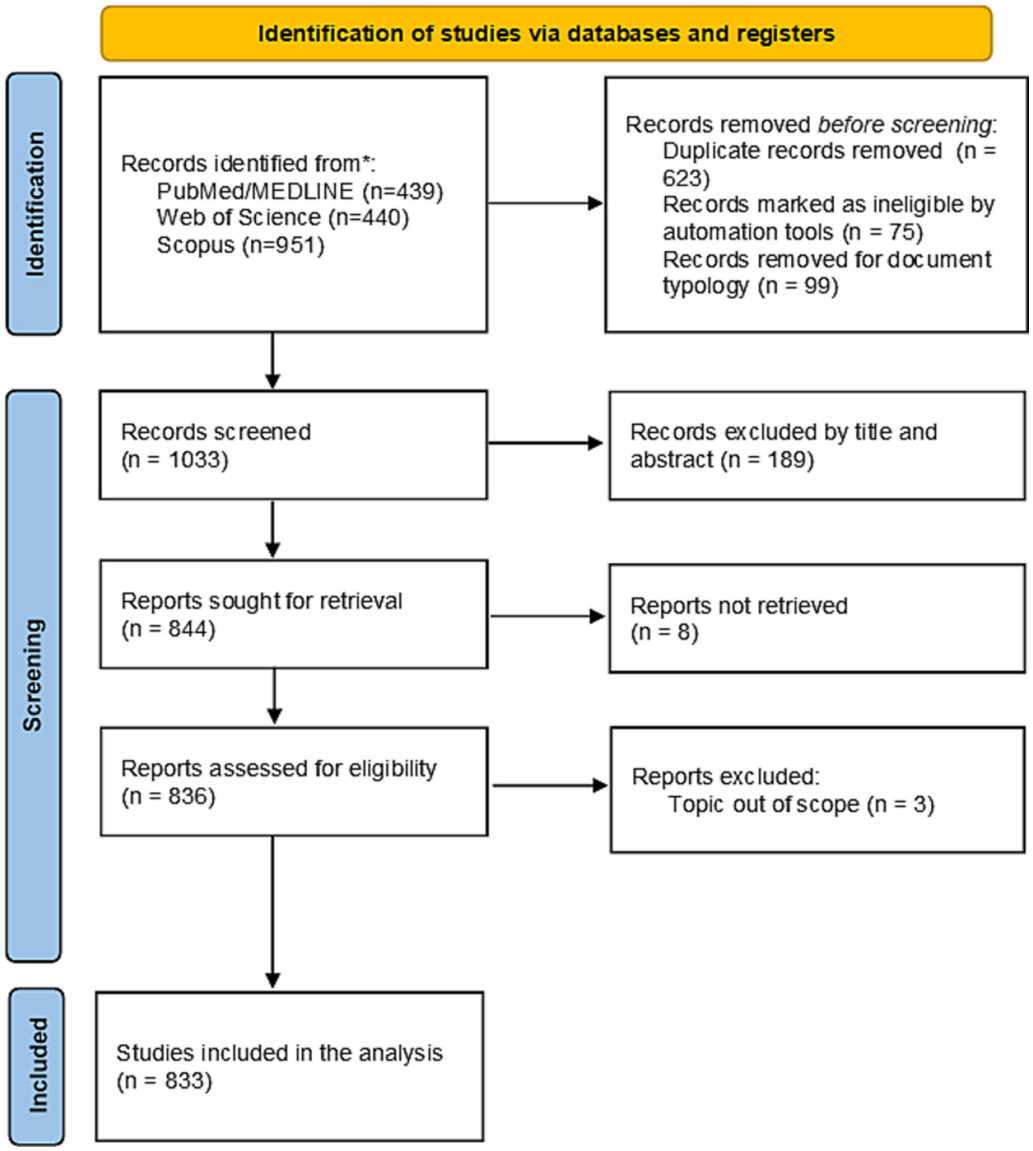
PRISMA flow diagram of study selection process for the review on deep brain stimulation in neurodevelopmental disorders.

Initially, 1830 results were identified, of which 833 were analyzed after the application of exclusion criteria (Figure 1). An examination of document types revealed that the majority of contributions were original research articles (51.6%) and review papers (35.7%). Other formats, such as letters (5.8%), notes (3.4%), and editorials (2.4%), represented a smaller fraction of the scientific discourse (Table 1). Notably, only 1.20% (*n* = 10) were identified as clinical trials, and 3.12% (*n* = 26) were classified as systematic reviews (Table 1). This finding underscores the predominance of observational or exploratory studies in the field and highlights a potential gap in high-level evidence synthesis and interventional research.

**Table 1:** Characteristics of the scientific publications on DBS in neurodevelopmental disorders, with a comparative subset focusing on functional performance and aggressiveness (1996–2025).

	Publications on deep brain stimulation and neurodevelopmental disorders (*N* = 833)	Publications on deep brain stimulation and neurodevelopmental disorders related to functional performance and mitigation of aggression (*N* = 52)
	*n* (%)	
Article type		
Original article	430 (51.62)	23 (44.23)
Review	297 (35.65)	26 (50)
Letter	48 (5.76)	2 (3.85)
Note	28 (3.36)	–
Editorial	20 (2.40)	1 (1.92)
Short Survey	10 (1.20)	–
Systematic reviews	26 (3.12)	4 (7.69)
Clinical trials	10 (1.20)	0
Geographic región*		
Europe & Central Asia	313 (37.58)	25 (48.08)
North America	259 (31.09)	11 (21.15)
East Asia & Pacific	96 (11.52)	5 (9.62)
Latin America & Caribbean	24 (2.88)	10 (19.23)
Middle East & North Africa	11 (1.32)	-
South Asia	9 (1.08)	1 (1.92)
Sub-Saharan Africa	2 (0.24)	–
Income group*		
High-income	615 (73.83)	39 (75)
Upper-middle income	88 (10.56)	12 (23.08)
Lower-middle income	11 (1.32)	1 (1.92)
Country*		
United States	223 (26.77)	8 (15.38)
Germany	85 (10.20)	3 (5.77)
France	53 (6.36)	0
China	48 (5.76)	2 (3.85)
Italy	47 (5.64)	5 (9.62)
Others	377 (45.27)	34 (65.38)
Funding		
Yes	302 (36.25)	13 (25)
No	531 (63.75)	39 (75)

*Classification according to the country of the corresponding author.

**Figure 2:**
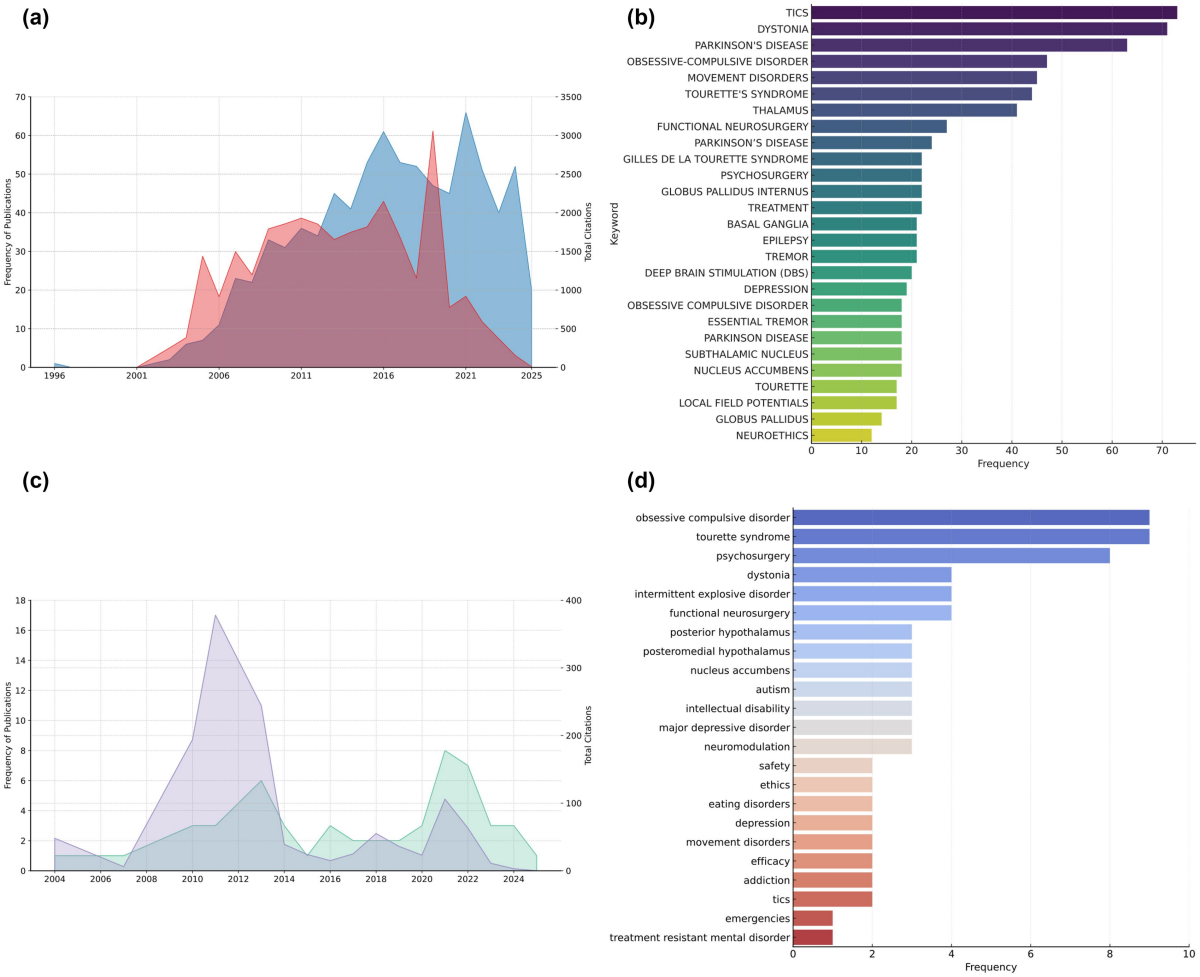
Trends and thematic characterization of scientific research on DBS in the context of neurodevelopmental disorders. **(a)** Annual trends in publication frequency (blue) and total citations (red) for studies addressing DBS in neurodevelopmental disorders, from 1996 to 2025 (*n* = 833). **(b)** Word cloud showing the most frequent keywords associated with DBS in neurodevelopmental disorders, excluding general descriptors to highlight specific thematic focuses. **(c)** Annual publication trends (green) and total citations (violet) for studies combining DBS and neurodevelopmental disorders with a focus on functional performance and aggressiveness (*n* = 52). **(d)** Most frequently occurring keywords in the subset of studies represented in panel **(c)**, reflecting the dominant research topics explored within this more specific thematic intersection.

Regarding publication trends over time, the number of articles has increased steadily, with notable growth after 2007 and a sharp rise observed in 2021 and early 2024 (Figure 2a). Citation counts also increased over the years, with earlier publications contributing more significantly to cumulative citations (Figure 2a), while more recent outputs have not yet reached their full citation potential. Importantly, it should be noted that the total number of articles for 2025 has not yet been finalized, as the year remains ongoing. This temporal limitation implies that both citation trends and publication volumes for the current year may still evolve, potentially altering the observed patterns over time.

### 3.2 Geographical and economic distribution

The analysis of geographic distribution by country showed that the United States (26.77%) led the scientific output, followed by Germany (10.2%), France (6.36%), and China (5.76%) (Table 1). These countries represent hubs of both technological innovation in neuromodulation and clinical research infrastructure. When grouped by geographic region, the majority of publications originated from Europe & Central Asia (37.58%), North America (31.09%), and East Asia & Pacific (11.52%) (Table 1). These regions also correspond to areas with robust research institutions and funding ecosystems. Similarly, in terms of income classification, high-income countries were the predominant contributors, accounting for over 75% of all publications. This finding underscores the existing disparities in global research capacity and access to advanced neuromodulators interventions such as DBS.

Concerning language of publication, English was overwhelmingly dominant, representing 92.8% of the total, followed by German (2.16%) and Chinese (1.08%) (Table 1). This reflects both the global orientation of the field and the predominance of English as the standard language of scientific communication.

### 3.3 Funding patterns

In terms of funding, approximately 36% of the publications explicitly reported receiving financial support (Table 1). This suggests that while a significant share of the research in this field is supported by grants, a considerable portion may still be conducted independently or without reported funding, which may have implications for research scope and methodological depth.

### 3.4 Thematic analysis

Thematic analysis based on keyword frequency revealed that current research on DBS in neurodevelopmental disorders has been primarily oriented in neurodegenerative diseases and clinical manifestations. Terms such as dystonia, Parkinson’s diseases, movement disorders, epilepsy, and tremor appeared most frequently (Figure 2b), suggesting a focus on populations with severe symptomatology or refractoriness to conventional treatments.

## 4 Scientific output on functional performance and aggressiveness in neurodevelopmental disorders treated with DBS

### 4.1 Baseline characteristics and publication trends

**Figure 3:**
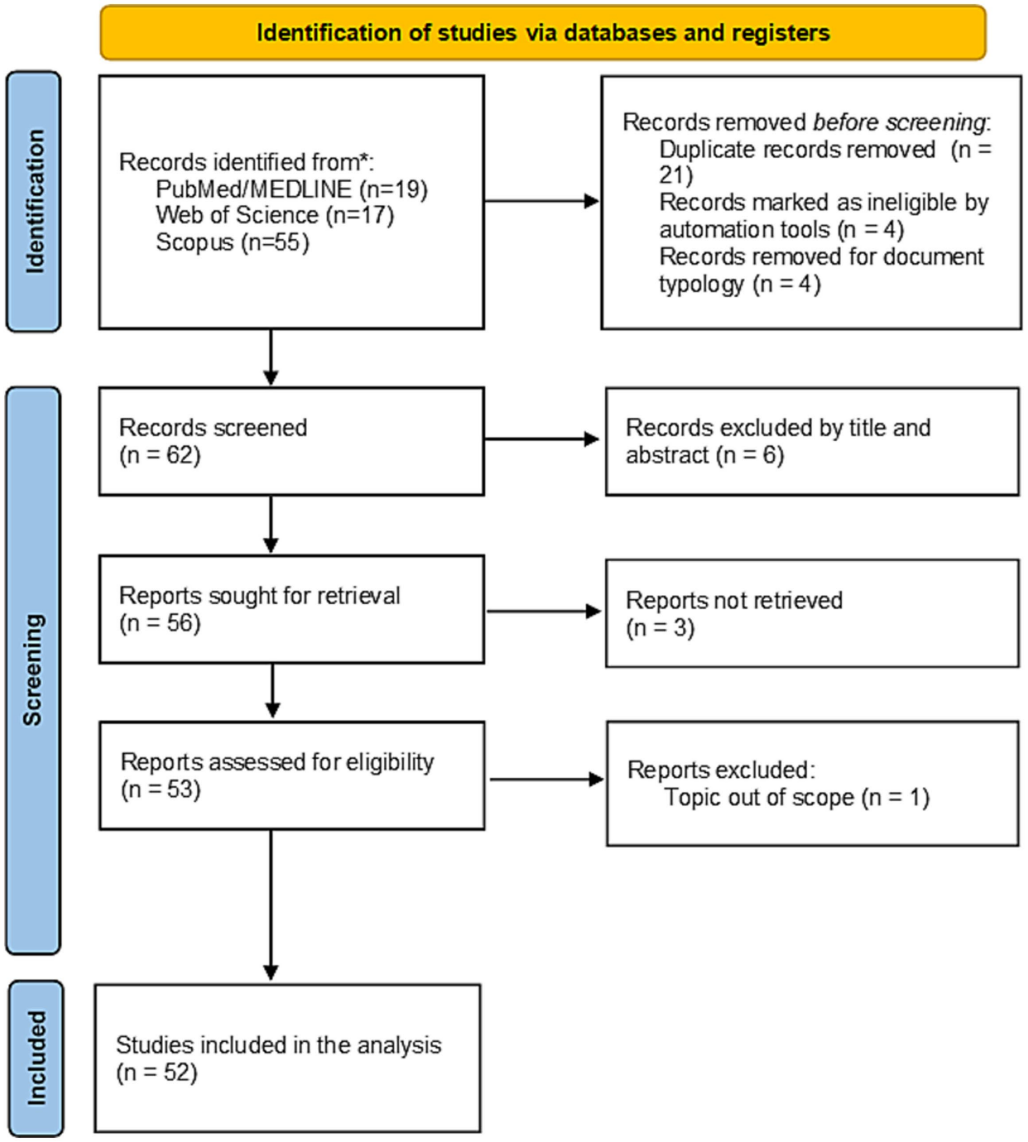
PRISMA flow diagram of study selection process for publications on deep brain stimulation in neurodevelopmental disorders addressing functional performance and/or aggressiveness.

Initially, 91 results were identified, of which 52 were analyzed after the application of exclusion criteria (Figure 3). Regarding document types, the majority were either review articles (*n* = 26; 50%) or original research articles (*n* = 23; 44.23%), while other types such as letters (3.9%) and editorials (1.9%) were less common (Table 1). No clinical trials were identified and only 4 (7.69%) systematic reviews were found (Table 1). Although slightly higher in relative terms, these values still reflect a limited presence of rigorous interventional and evidence synthesis studies in this focused research niche.

In terms of temporal trends, the number of publications showed a modest increase over time, with publication activity peaking in 2021 (Figure 2c). Citation accumulation remained relatively low across the dataset, consistent with the recent emergence of many of the included studies (Figure 2c). The total citation count per year remains limited, with earlier years contributing marginally due to the recent character of this research focus.

### 4.2 Funding patterns

Regarding funding, 25% explicitly reported having received financial support, while the remaining 75% either lacked funding or did not disclose it (Table 1). This suggests a non-balanced distribution between funded and non-funded scientific output, which may reflect early-stage exploration of the topic by both institutional and independent researchers.

### 4.3 Geographical and economic distribution

Geographically, the United States was the most represented country (15.38%), followed by United Kingdom (13.46%), Colombia (11.54%), Netherlands (9.62%), and China (9.62%) (Table 1). This distribution mirrors broader trends in neuromodulation research and highlights key hubs for scientific output. When grouped by world regions, Europe & Central Asia (48.08%) and North America (21.15%) accounted for the largest shares of publications, followed by Latin America & Caribbean (19.23%) (Table 1). In terms of income classification, the vast majority of contributions originated from high-income countries (75%), with minimal participation from upper-middle-income economies (23.08%) and none from low-income countries (Table 1).

### 4.4 Thematic analysis

In the focused subset, keywords remained predominantly diagnosis-driven, with obsessive compulsive disorders, Tourette syndrome, and psychosurgery among the most recurrent terms. While some outcome-oriented constructs such as intellectual disability, safety and efficacy were represented (Figure 2d), the overall thematic orientation still favored the clinical profile of patients over the specific functional or behavioral outcomes targeted by DBS as expected in neuroscientific research, English was the dominant language of publication, representing 92.31% of the documents analyzed, followed by Japanese (3.85%) (Table 1).

## 5 Discussion

Despite the growing scientific interest in DBS for neurodevelopmental disorders, our findings reveal a notable underrepresentation of high-level evidence across both the general and focused analyses. Only 1.20% of the total publications on DBS in neurodevelopmental disorders were clinical trials, and 3.12% consisted of systematic reviews. This limitation was even more intense in the sub-analysis, where no clinical trials were identified, and there are only four systematic reviews. These results demonstrate that the available evidence that might be required to justify interventions in this field remains disproportionately low, relative to the translational relevance and clinical expectations (Shea et al., 2025).

The near-absence of clinical trials severely limits the development of evidence-based guidelines for DBS in neurodevelopmental disorders. This scarcity not only affects clinical decision-making but also hampers innovation in outcome measurement, safety profiling, and patient selection criteria. Compared to the well-established evidence base for pharmacological and behavioral therapies in neurodevelopmental disorders, DBS remains largely experimental. This contrast further underscores the need for high-quality interventional research in this domain.

This gap suggests that most contributions to the field remain exploratory or observational in nature, often lacking the methodological rigor needed to support clinical decision-making or guideline development (Moosapour et al., 2021). The scarcity of structured evidence synthesis impairs the capacity of the field to identify replicable mechanisms of action, patient selection criteria, or optimal stimulation parameters (Alqaidoom et al., 2023). If DBS is to fulfill its promise in reshaping neurodevelopmental care, particularly in challenging behavioral contexts, the field must embrace a more systematic, trial-based, and review-driven approach to knowledge production (Moosapour et al., 2021).

These findings also highlight persistent inequalities in the global distribution of research on DBS in neurodevelopmental disorders. High-income countries in North America and Western Europe continue to dominate scientific production, while contributions from low- and middle-income regions remain rare or nonexistent. This imbalance, consistently observed in both datasets, raises concerns about the generalizability of findings, as well as the ethical and practical implications of excluding vast portions of the global population from innovation pipelines (Naqvi et al., 2024).

However, Colombia (11.54%) emerged as a key contributor in the focused subset, surpassing traditional research leaders like France and Germany. Notably, most studies from Colombia were unfunded, highlighting the potential of emerging research hubs in Latin America despite limited financial resources. High-income countries accounted for 75% of the studies; however, only 25% of the subset received reported funding, suggesting limited financial support even in dominant regions. To address these geographic disparities, south–south scientific collaborations, inclusion in global funding mechanisms, and the development of regional neurotechnological networks are essential. Strengthening institutional capacity in low- and middle-income countries could help bridge the translational divide.

In the context of neurodevelopmental disorders, which frequently intersect with social vulnerability, healthcare disparities, and limited access to advanced interventions (Patrick et al., 2025), this exclusion is particularly problematic (Raouafi et al., 2018). Without proactive efforts to promote inclusivity in research funding, infrastructure, and authorship, the field risks deepening existing inequities in access to care and technological innovation (Lavis et al., 2010). Multicenter collaborations, global funding consortia, and south–south cooperation mechanisms should be encouraged to democratize both knowledge production and therapeutic implementation (Saloojee and Pettifor, 2024).

Finally, this study demonstrates how scientometrics approaches can play a pivotal role in strategically guiding the future of DBS research. By mapping publication dynamics, keyword evolution, and thematic densities, we were able to identify areas of saturation, stagnation, and growth (Lozada-Martinez et al., 2025a,b). More importantly, we were able to expose critical blind spots, such as the lack of focus on functional performance and aggressiveness, and quantify their magnitude within the broader research ecosystem. In addition to mapping thematic trends, scientometrics methods could be leveraged to uncover authorship disparities and structural silos within research networks, revealing where cross-disciplinary integration is lacking.

This type of meta-research provides a data-informed foundation for shaping research agendas, prioritizing funding calls, and aligning clinical research with patient-centered outcomes (Lozada-Martinez et al., 2025c; Stevens and Laynor, 2023). As DBS continues to evolve from an experimental therapy to a potentially mainstream intervention in neurodevelopmental care, scientometrics will become increasingly necessary to avoid duplication, accelerate translation, and ensure that clinical relevance is not eclipsed by technological fascination (Dockès et al., 2020). Importantly, the strategic use of this evidence is essential for informing regulatory decisions, clinical guidelines, and public investment priorities, ensuring that innovation is guided by measurable benefits for patients. Future research should incorporate outcomes that go beyond symptom control, placing greater emphasis on quality of life, functional autonomy, and patients’ ability to self-manage their care (Benedetti-Isaac et al., 2021; Benedetti-Isaac et al., 2023; Herrera-Pino et al., 2024).

## 6 Limitations

This study has limitations that should be acknowledged. First, while the search strategy was detailed and adapted for three major databases, the full search strings were only exemplified for Scopus, with the assumption that equivalent descriptors were used in PubMed and Web of Science. Although this approach ensured consistency, we did not provide a supplementary table listing every search string due to space constraints.

The process of data screening and extraction, although conducted independently by two researchers and validated by consensus, did not include formal inter-rater agreement statistics (e.g., kappa coefficients). This limits the quantification of potential reviewer discrepancies, although the risk of bias was minimized through cross-validation.

The categorization of countries under a collective “Others” label in Table 1, while necessary due to formatting and frequency distribution constraints, may obscure the visibility of emerging contributors such as Colombia. This editorial decision affects the granularity of the geographic analysis and may limit the full interpretation of global asymmetries.

The study is inherently descriptive and relies on bibliometric metadata, which does not provide insight into study quality, clinical outcomes, or methodological rigor beyond what is reported in indexed records. Finally, the findings are disproportionately influenced by high-income countries, reflecting long-standing imbalances in global research ecosystems. Although this was one of the gaps we aimed to highlight, the limited participation of lower-income countries reduces the generalizability of trends and underscores the need for more inclusive and equitable research efforts.

Despite these limitations, the study provides a valuable meta-research perspective on the structural gaps in DBS research for neurodevelopmental disorders and offers actionable insights to guide future investigations, including mental health (Acuña Rodriguez et al., 2025; Blanco-Teherán et al., 2022).

## 7 Conclusion

This study reveals notable gaps in the scientific exploration of DBS for neurodevelopmental disorders, particularly in relation to functional performance and the mitigation of aggressiveness. Although research output has grown consistently, only a small fraction of studies focus on these critical clinical outcomes. Evidence from clinical trials and systematic reviews remains scarce, and most research continues to center on diagnostic categories rather than on patient-centered functional outcomes. These patterns suggest a disconnect between current scientific priorities and the therapeutic potential of DBS. Addressing these gaps will require a deliberate shift in research agendas toward outcomes that reflect real-world needs and advance the impact of this intervention in neurodevelopmental care.

Future studies should prioritize trial-based designs, incorporate outcome-oriented endpoints such as quality of life and functional autonomy, and promote equitable international research partnerships that include underrepresented regions. These measures are essential for aligning funding policies with real-world patient needs and advancing global neurotechnological equity.

## Data Availability

The original contributions presented in the study are included in the article/supplementary material, further inquiries can be directed to the corresponding authors.
